# Genetic mechanisms and correlated risk factors of antimicrobial-resistant ESKAPEE pathogens isolated in a tertiary hospital in Malaysia

**DOI:** 10.1186/s13756-021-00936-5

**Published:** 2021-04-23

**Authors:** Soo Tein Ngoi, Chun Wie Chong, Sasheela Sri La Sri Ponnampalavanar, Soo Nee Tang, Nuryana Idris, Kartini Abdul Jabar, Michael J. Gregory, Tupur Husain, Cindy Shuan Ju Teh

**Affiliations:** 1 Department of Medical Microbiology, Faculty of Medicine, Universiti Malaya, 50603 Kuala Lumpur, Malaysia; 2 School of Pharmacy, Monash University Malaysia, 47500 Bandar Sunway, Selangor, Malaysia; 3 Department of Medicine, Faculty of Medicine, Universiti Malaya, 50603 Kuala Lumpur, Malaysia; 4 United States Naval Medical Research Unit Two (NAMRU-2), Phnom Penh, Cambodia

**Keywords:** AMR-conferring genes, Minimum inhibitory concentration, Molecular epidemiology, Multidrug-resistant organisms, Nosocomial infections, Risk factors analysis

## Abstract

**Background:**

Knowledge on the epidemiology, genotypic and phenotypic features of antimicrobial-resistant (AMR) ESKAPEE pathogens (*Enterococcus* *faecium*, *Staphylococcus* *aureus*, *Klebsiella* *pneumoniae*, *Acinetobacter* *baumannii*, *Pseudomonas* *aeruginosa*, *Enterobacter* spp., and *Escherichia* *coli*) and their association with hospital-acquired infections (HAIs) are limited in Malaysia. Therefore, we evaluated the AMR features and resistance mechanisms of the ESKAPEE pathogens collected in a tertiary hospital located in the capital of Malaysia.

**Methods:**

A total of 378 AMR-ESKAPEE strains were obtained based on convenience sampling over a nine-month study period (2019–2020). All strains were subjected to disk diffusion and broth microdilution assays to determine the antimicrobial susceptibility profiles. Polymerase chain reaction (PCR) and DNA sequence analyses were performed to determine the AMR genes profiles of the non-susceptible strains. Chi-square test and logistic regression analyses were used to correlate the AMR profiles and clinical data to determine the risk factors associated with HAIs.

**Results:**

High rates of multidrug resistance (MDR) were observed in *A. baumannii*, *K. pneumoniae*, *E. coli*, and *S. aureus* (69–89%). All organisms except *E. coli* were frequently associated with HAIs (61–94%). Non-susceptibility to the last-resort drugs vancomycin (in *Enterococcus* spp. and *S. aureus*), carbapenems (in *A. baumannii*, *P. aeruginosa*, and *Enterobacteriaceae*), and colistin (in *Enterobacteriaceae*) were observed. Both *A. baumannii* and *K. pneumoniae* harbored a wide array of extended-spectrum β-lactamase genes (*bla*_TEM_, *bla*_SHV_, *bla*_CTX-M_, *bla*_OXA_). Metallo-β-lactamase genes (*bla*_VEB_, *bla*_VIM_, *bla*_NDM_) were detected in carbapenem-resistant strains, at a higher frequency compared to other local reports. We detected two novel mutations in the quinolone-resistant determining region of the *gyr*A in fluoroquinolone-resistant *E. coli* (Leu-102-Ala; Gly-105-Val). Microbial resistance to ampicillin, methicillin, and cephalosporins was identified as important risk factors associated with HAIs in the hospital.

**Conclusion:**

Overall, our findings may provide valuable insight into the microbial resistance pattern and the risk factors of ESKAPEE-associated HAIs in a tertiary hospital located in central Peninsular Malaysia. The data obtained in this study may contribute to informing better hospital infection control in this region.

**Supplementary Information:**

The online version contains supplementary material available at 10.1186/s13756-021-00936-5.

## Background

The antimicrobial resistance (AMR) phenomenon among clinically important human pathogens remains a major threat to public health worldwide. The global emergence and spread of AMR organisms have been documented and are often associated with increasing incidences of treatment failure and adverse clinical outcomes [1]. The World Health Organization (WHO) reported a significant increase in the mortality rates and intensive care unit (ICU) admission rates of patients infected by AMR organisms, based on a systematic review on drug-resistant *Escherichia coli*, *Klebsiella pneumoniae*, and *Staphylococcus aureus* [1]. The emergence of bacteria that are resistant to multiple antimicrobial agents has gained notoriety as an important global public health issue [2]. Infections caused by multidrug-resistant organisms (MDROs) are reported everywhere in the world, compromising our ability to treat infectious diseases. Due to the impact of MDROs on public health, WHO released a list of the world’s most dangerous superbugs in February 2017 [3]. The ESKAPEE pathogens (*Enterococcus faecium, Staphylococcus aureus, Klebsiella pneumoniae, Acinetobacter baumannii, Pseudomonas aeruginosa, Enterobacter* species*,* and *Escherichia coli*) are listed as organisms of critical importance due to their MDR nature and increased resistance to last-resort drugs, consequently requiring urgent development of new antibiotics [3].

Antimicrobial resistance occurs naturally over time through genetic mutations and exchanges, although these genetic events usually take place at low frequencies [4]. The process of AMR emergence, dissemination, and persistence among bacteria, however, has been accelerated due to the overuse or misuse of antimicrobials in the hospital settings, livestock farming, and aquaculture industry [5]. The development of acquired resistance in commensals or previously non-resistant bacteria can be attributed to chromosomal mutations or acquisition of external genetic determinants of resistance via horizontal gene transfer (HGT) within a reservoir [6]. The microbiomes of humans and animals, as well as environmental sources, frequently serve as the reservoirs for HGT among the bacterial cells. *Escherichia coli, Klebsiella pneumoniae, Enterobacter* spp.*,* and *Enterococcus* spp*.* are intestinal commensals that were once considered relatively harmless, have now evolved into MDROs that are responsible for various healthcare-associated infections (HCAIs) [7, 8]. In addition, the increase in the number of infections caused by other MDR opportunistic pathogens, such as *Staphylococcus aureus*, *Acinetobacter baumannii,* and *Pseudomonas aeruginosa* in hospitals has also been widely observed in Asia [9].

HCAI is defined as an infection acquired by a patient under medical care in the hospital or other healthcare facility, which was absent at the time of admission [10]. HCAI represents the most frequent adverse event in healthcare delivery worldwide. In Malaysia, the hospital-wide prevalence of HCAIs is rated at 14%, two-fold higher than neighboring countries Thailand (6.5%) and Indonesia (7.1%) [10]. HCAIs often pose a significant threat to hospitalized individuals and further complicate infection management in healthcare settings, particularly in critical care units where patients have an increased risk of infection due to their underlying diseases or comorbidity predispositions, impaired immunity, and exposure to multiple invasive devices such as catheters [11]. HCAIs caused by MDROs are more difficult to treat and result in a significant increase in mortality rates and financial losses for the health system in a country [12]. The common practice of initial broad-spectrum antibiotics treatment often selects for MDROs persistence in the healthcare settings, leading to the development of severe HCAIs in institutionalized patients [11].

Effective infection prevention and control measures are critical in limiting the dissemination of drug-resistant bacteria in healthcare settings, and thus preventing HCAI outbreaks. However, a lack of adequate systems and research support has hampered the ability in many healthcare settings to effectively implement comprehensive infection control strategies [13]. In order to establish a monitoring pathway for bacterial transmissions and infections, information such as the resistance mechanisms should be linked with the strains phenotypic profiles as well as the host characteristics. Molecular surveillance, and in particular the surveillance of resistance genes detection, is necessary for hospital infection control, through the detection of emerging pathogens and monitoring of AMR trends for better patient management. Further, understanding of the AMR mechanisms would also aid in the prediction of underlying or unknown resistance mechanisms, susceptibility to other antimicrobial agents, and would contribute to the development of new drug or therapeutics to combat MDROs infection [14].

In this study, we aimed to investigate the genotypic and phenotypic features of drug-resistant ESKAPEE pathogens in a tertiary hospital to identify the AMR trends and molecular epidemiology of the organisms throughout the study period. Microbiological data were associated with host characteristics and clinical factors to provide a comprehensive insight into rational treatment strategies and infection control measures in Malaysian hospitals.

## Materials and methods

### Bacterial strains collection and identification

A total of 378 clinical, non-repeat bacterial strains were prospectively collected from the diagnostic laboratory of the University of Malaya Medical Centre (UMMC), a tertiary hospital located in Kuala Lumpur Malaysia, within a nine-month study period (October 2019–July 2020). The ESKAPEE pathogens investigated in this study include *Enterococcus* spp., *Staphylococcus aureus* (*S. aureus*), *Klebsiella pneumoniae* (*K. pneumoniae*), *Acinetobacter baumannii* (*A. baumannii*)*, Pseudomonas aeruginosa* (*P. aeruginosa*)*, Enterobacter* spp., and *Escherichia coli* (*E. coli*). All bacterial strains (n = 54 per species) were first identified based on conventional culture method and automated microbial identification VITEK® 2 system (bioMérieux, Marcy-l'Étoile, France), during routine microbiological investigations at the hospital’s diagnostic laboratory. The identity of the bacterial strains was further confirmed by polymerase chain reaction (PCR) detection of genus/species-specific genes from the boiled lysate of bacterial cells, prior to further phenotypic and genotypic analyses. The PCR primers and reaction mixture compositions were adapted from published studies (Additional file 1: Table S1). Minor modifications were made to the thermal-cycling parameters (Additional file 1: Table S1). Agarose gel electrophoresis was used to visualize the presence of amplicons in the PCR products. The agarose gel (1% w/v) was stained with GelRed® Nucleic Acid Gel Stain (Biotium, Fremont, USA) and viewed using a blue light transilluminator.

### Antimicrobial susceptibility testing of the bacterial strains

The antimicrobial susceptibility profiles of the confirmed bacterial strains were determined by the hospital’s diagnostic laboratory using VITEK® 2 system. All ESKAPEE strains selected for further investigation were resistant to at least one antibiotic based on VITEK® 2 analyses. The antimicrobial susceptibility phenotypes of the bacterial strains against selected agents were further confirmed via the Kirby-Bauer disk diffusion method based on Clinical and Laboratory Standards Institute (CLSI) recommendations [15]. The zones of inhibition for the tested antimicrobial agents were interpreted according to CLSI guidelines. Bacterial strains showing reduced susceptibility or resistant to selected antimicrobial agents of interest were further subjected to minimum inhibitory concentration (MIC) testing using broth microdilution method based on CLSI guidelines. The median MIC value is indicated as MIC_50_. The MIC required to inhibit the growth of 90% of the bacterial strains is indicated as MIC_90_.

### Characterization of the antimicrobial resistance gene profiles of the ESKAPEE pathogens

Based on the antimicrobial susceptibility test results, all ESKAPEE strains that were non-susceptible to the tested antimicrobial agents were further subjected to molecular analysis to determine the AMR gene profiles. PCR assays were conducted using established primer sequences adapted from published studies (Additional file 1: Table S2). AMR genes encoding for vancomycin resistance (*van*AB), aminoglycoside-modifying enzymes (*aac*, *aad*, *ant*, and *aph* variants), methicillin resistance (*mec*A), erythromycin resistance (*erm*ABC), quinolone/fluoroquinolone resistance (*gyr*AB, *par*CE, and *qnr*ABC), and beta-lactamases (TEM, SHV, CTX-M, OXA, VIM, IMP, NDM, KPC, PER, VEB, and SIM) were selectively screened based on bacterial species. The PCR conditions used in this study were optimized for a lower reaction volume. All targeted genes were individually amplified in a 25 µL reaction mixture consisting of 1 × PCR buffer, 1.5 mM MgCl_2_, 100 µM deoxynucleoside triphosphates (dNTPs), 1 U *Taq* polymerase (Promega, Madison, USA), and approximately 150 ng of DNA template obtained from the boiled lysate of bacterial cell suspension. Working concentrations of the primers for the targeted genes or genetic regions are shown in Additional file 1: Table S2. PCR thermal cycling conditions for all reactions included an initial denaturation at 95 °C (5 min), followed by 30 cycles of denaturation at 94 °C (40 s), annealing at 48–68 °C (40 s) (Additional file 1: Table S2), and extension at 72 °C (1 min). The PCR cycles ended with a final extension at 72 °C for 7 min. Agarose gel electrophoresis was used to visualize the presence of a PCR amplicon. For the identification of gene variants, the PCR products were purified using MEGAquick-spin™ Plus Total Fragment DNA Purification Kit (iNtRON Biotechnology, Gyeonggi-do, Korea). Next, the purified amplicons were subjected to DNA sequencing by a commercial sequencing service provider (First BASE Laboratories, Petaling Jaya, Malaysia), and the sequences obtained were analyzed using National Center for Biotechnology Information (NCBI) Basic Local Alignment Search Tool (BLAST) to identify the gene variant. In order to determine the position of base substitutions in the gyrase (*gyr*AB) and topoisomerase IV (*par*CE) genes, the amplicon sequences were aligned and compared with the reference gene sequences in *E. coli* strain ATCC 25922 (NCBI GenBank accession number CP037449) by using the Molecular Evolutionary Genetics Analysis (MEGA) X software [16].

### Patient data collection and correlation analysis

Relevant patient and clinical data including basic demographic information (age, gender, and ethnicity), admission date, bacterial isolation date, and type of specimen source were extracted from the clinical database. The mode of acquisition of infection, i.e. community-acquired (CA) or hospital-acquired (HA), was inferred based on the patient’s admission date and bacterial isolation date. Infection is determined as HA if the patient developed signs of infection and a positive bacterial culture was obtained at 48 h or more post-admission. Descriptive statistics are expressed as percentage unless otherwise stated. Categorical variables were expressed as percentages and compared using the Chi-square test. Variables with a univariate test value of less than 0.1 (p-value) were included in a multivariate analysis using a logistic regression model. Odds ratios (OR) and 95% confidence intervals (CI) were calculated.

## Results:

The clinical features of all 378 ESKAPEE strains collected during study period are summarized in Table 1. The AMR rates and MIC ranges of selected antimicrobial agents, as well as the prevalence of AMR genes for each bacterial species, are individually reported in this section. The detailed strain profiles for each of the ESKAPEE organisms are provided in Additional file 1: Table S3–S9.

**Table 1:** Summary of the clinical features of ESKAPEE strains (n = 378) examined in this study

	*Enterococcus* spp. (n = 54)	*S. aureus* (n = 54)	*K. pneumoniae* (n = 54)	*A. baumannii* (n = 54)	*P. aeruginosa* (n = 54)	*Enterobacter spp.* (n = 54)	*E. coli* (n = 54)
	(n) (%)	(n) (%)	(n) (%)	(n) (%)	(n) (%)	(n) (%)	(n) (%)
*Age range*							
< 18 years	4 (7)	3 (6)	5 (9)	1 (2)	8 (15)	5 (9)	4 (7)
18—59 years	24 (45)	20 (37)	18 (33)	25 (46)	18 (33)	20 (37)	17 (32)
≥ 60 years	26 (48)	31 (57)	31 (58)	28 (52)	28 (52)	19 (35)	33 (61)
*Gender*							
Male	33 (61)	31 (57)	35 (65)	37 (69)	30 (56)	36 (67)	26 (48)
Female	21 (39)	23 (43)	19 (35)	17 (31)	24 (44)	18 (33)	28 (52)
*Ethnicity*							
Malay	18 (33)	21 (39)	16 (30)	14 (26)	20 (37)	19 (35)	14 (26)
Chinese	17 (31)	17 (31)	23 (43)	15 (28)	21 (39)	18 (33)	26 (48)
Indian	16 (30)	16 (30)	11 (20)	22 (40)	12 (22)	15 (28)	11 (20)
Others	3 (6)	0 (0)	4 (7)	3 (6)	1 (2)	2 (4)	3 (6)
*Mode of acquisition*							
Hospital acquired	36 (67)	35 (65)	38 (72)	51 (94)	34 (63)	33 (61)	25 (46)
Community acquired	18 (33)	19 (35)	15 (28)	3 (6)	20 (37)	21 (39)	29 (54)
*Specimen source*							
Respiratory sites	0 (0)	12 (22)	18 (33)	37 (69)	30 (56)	26 (48)	7 (13)
Body fluids (blood, tissue fluids, urine, etc.)	37 (69)	20 (37)	27 (50)	7 (13)	17 (31)	6 (11)	32 (59)
Tissues and swabs	17 (31)	22 (41)	9 (17)	10 (18)	7 (13)	22 (41)	15 (28)
*Hospital admission record*							
Critical/Intensive care units	7 (13)	1 (2)	10 (19)	27 (50)	6 (11)	8 (15)	6 (11)
Medical wards	44 (81)	51 (94)	41 (77)	27 (50)	41 (76)	40 (74)	43 (80)
Outpatient	3 (6)	2 (4)	2 (4)	0 (0)	7 (13)	6 (11)	5 (9)
*Multidrug resistance*							
MDR	2 (4)	37 (69)	47 (87)	48 (89)	11 (20)	6 (11)	37 (69)
Non-MDR	52 (96)	17 (31)	7 (13)	6 (11)	43 (80)	48 (89)	17 (31)

## *Enterococcus* spp.

Among the 54 *Enterococcus* spp. strains examined in this study, *E. faecalis* (43%) and *E. faecium* (41%) made up the major portion of the strains collection. Other *Enterococcus* species obtained from the clinical specimens include *avium* (n = 1), *gallinarum* (n = 1), *hirae* (n = 2), and *raffinosus* (n = 3). Majority of the *Enterococcus* spp. strains were associated with HA infections (67%) and 19% of which occurred in patients admitted to the intensive care units (ICUs). A considerable subset of the *Enterococcus* spp. (39%) were highly resistant to ampicillin with MIC values ranging from 64 µg/mL to 256 µg/mL and above (MIC_50_ = 256 µg/mL). The majority of the strains (87%) were susceptible to vancomycin. Six vancomycin-resistant enterococci (VRE) strains were identified in this study (Table 2). The majority of the VRE (67%) showed a low level of resistance to vancomycin (MIC ≤ 32 µg/mL). Most of the VRE (83%) were isolated from blood and bone specimens of the patients, indicating the invasive nature of the organisms. Half of the VRE were associated with HA infections. All *Enterococcus* strains exhibited a streptomycin-resistant phenotype with a high level of resistance (MIC_50_ = 128 µg/mL). High-level aminoglycoside resistance (HLAR) was indicated in 39% of the *Enterococcus* spp., whereby the strains endured a high concentration of streptomycin at 1000 µg/mL in the growth medium.

**Table 2:** *Enterococcus* strains with resistant or reduced susceptibility phenotypes to vancomycin

Study code	*Enterococcus* species	Type of infection	Specimen	Vancomycin		AMR genes
				Susceptibility	MIC (µg/mL)	
ENC/UM/01	*E. faecium*	HA	Blood	R	32	*van*A*, van*B
ENC/UM/32	*E. faecium*	HA	Swab	R	> 256	*van*A*, van*B
ENC/UM/33	*E. faecalis*	CA	Bone	R	32	*van*B
ENC/UM/46	*E. faecalis*	HA	Tissue	I	16	*van*B
ENC/UM/52	*E. faecalis*	CA	Bone	R	32	-
ENC/UM/54	*E. faecalis*	HA	Blood	R	32	*van*B
ENC/UM/57	*E. faecalis*	CA	Blood	R	> 256	*van*B

The *Enterococcus* spp. strains were screened for the presence of six aminoglycoside resistance-conferring gene variants in this study. The most prevalent aminoglycoside-modifying enzyme (AME) gene was *ant(4′)-Ia* (22%), followed by *aph(2″)-Ic* (15%), and *aac(6′)-Ie-aph(2″)-Ia* (7%). The AME genes *aph(2″)-Ib*, *aph(2″)-Id*, and *aph(3′)-IIIa* were absent among the strains. All except one VRE strains harbored the *van*B gene (Table 2). Two HA-VRE strains, one obtained from a swab sample and one from a blood sample, carried both *van*A and *van*B genes. Interestingly, the presence of both *van* genes in the VRE strains does not necessarily indicate a high level of vancomycin resistance. One of the VRE strains harboring both *van* genes was highly resistant to vancomycin (MIC ≥ 256 µg/mL), while the other showed only low-level resistance (MIC = 32 µg/mL). One *E. faecalis* strain with reduced susceptibility to vancomycin (MIC = 16 µg/mL) harbored a *van*B gene.

### *Staphylococcus aureus*

The *S. aureus* strains examined in this study comprised of majority methicillin-resistant *S. aureus* (MRSA) (85%) and only eight methicillin-susceptible *S. aureus* (MSSA) (15%). The majority of *S. aureus* (65%) caused HA infections. The MSSA strains were found to be more commonly associated with CA infections compared to their methicillin-resistant counterpart. The *S. aureus* was mostly MDR (69%). The MDR phenotype was especially common among the MRSA (80%), while all MSSA strains remained susceptible to most of the antimicrobial agents tested. The *S. aureus* strains were largely resistant to penicillin (98%). All strains were susceptible to linezolid, rifampicin, and vancomycin. The MIC values of vancomycin ranged from less than 0.5 µg/mL to 1 µg/mL (MIC_50/90_ = 0.5/1 µg/mL).

The identity of the MRSA strains was confirmed by both oxacillin and cefoxitin (agent for the surrogate test) resistance. All MRSA strains showed a moderate or high level of resistance to oxacillin, with MIC values ranging from 16 µg/mL to 256 µg/mL and above (MIC_50_ = 256 µg/mL). The presence of the *mec*A gene in all MRSA strains was confirmed by molecular detection of the gene using the PCR method. The majority of the *S. aureus* strains (69%) were identified as clindamycin-resistant, although most of which were considered inducible clindamycin resistance (73%) as indicated by erythromycin resistance. The erythromycin resistance-conferring gene, *erm*C, was identified in all *S. aureus* strains that exhibited erythromycin-resistant phenotype (n = 37).

### *Acinetobacter baumannii*

The *A. baumannii* strains examined in this study were mostly associated with HA infections (94%). Half of the *A. baumannii* strains were isolated from ICUs, including general, cardiac, and neonatal ICUs. The majority of the *A. baumannii* were obtained from clinical specimens related to the respiratory sites (69%). The *A. baumannii* were mostly MDR (89%). All strains were resistant to imipenem, with MIC values ranging from 32 µg/mL to 128 µg/mL (MIC_50_ = 128 µg/mL). Therefore, all strains were identified as carbapenem-resistant *A. baumannii* (CRAB). The majority of the CRAB strains were highly resistant to amikacin (87%; MIC ≥ 256 µg/mL), and all were highly resistant to piperacillin-tazobactam (MIC ≥ 512/4 µg/mL). Colistin resistance was observed in 19% of the CRAB strains, with a low or moderate level of resistance (8 µg/mL ≤ MIC ≤ 32 µg/mL).

The most encountered carbapenemase and extended-spectrum β-lactamase (ESBL) genes in our collection of CRAB strains were *bla*_OXA-23_ (96%), *bla*_OXA-51_ (91%), and *bla*_TEM_ (74%). Four strains harbored *bla*_VEB_, all of which were isolated from ICU patients with HA-pneumonia. The carbapenemase and ESBL genes present in the CRAB strains are summarized in Table 3. Other β-lactamase genes (*bla*_IMP_, *bla*_NDM_, *bla*_VIM_, *bla*_SHV_, *bla*_CTX-M_, *bla*_PER_, and *bla*_SIM_) were absent from the *A. baumannii* strains collected in this study.

**Table 3:** β-lactamase genes profile of the carbapenem-resistant *A. baumannii* strains

Cabapenemase/ESBL genes profile	Number of strains
*bla*_OXA-23_*, bla*_OXA-51_*, bla*_TEM_	33
*bla*_OXA-23_*, bla*_OXA-51_	12
*bla*_OXA-23_*, bla*_OXA-51_*, bla*_TEM_*, bla*_VEB_	3
*bla*_OXA-23_*, bla*_TEM_	3
*bla*_OXA-23_*, bla*_OXA-51_*, bla*_VEB_	1
*bla*_TEM_	1

The most common AME gene that contributed to amikacin resistance in CRAB strains was the *aad*B gene (49%). The *aac*A4, *aac*C1, and *aac*C2 genes were present in only 4% of the amikacin-resistant strains. The *A. baumannii* strains that harbored a combination of two AME genes (one strain with both *aac*C1 and C2; one with *aac*A4 and *aad*B; one with *aac*C1 and *aad*B) were found associated with HA-pneumonia in the ICUs.

### *Pseudomonas aeruginosa*

The majority of the *P. aeruginosa* (63%) examined in this study were associated with HA infections, six of which were isolated from patients admitted into ICU. More than half of the strains (56%) were isolated from clinical specimens associated with the respiratory tract. The majority of the strains (80%) showed a non-MDR phenotype. Although MDR phenotype was relatively uncommon among the *P. aeruginosa* compared to the MRSA and CRAB strains examined in this study, resistance to the first-line antibiotics in the aminoglycoside (amikacin), third generation cephalosporins (ceftazidime and cefotaxime), fluoroquinolone (ciprofloxacin), and carbapenem (imipenem and meropenem) families was at an alarming rate. Amikacin resistance was observed in 17% of the *P. aeruginosa* strains. One-fifth of the *P. aeruginosa* strains were ciprofloxacin-resistant (20%), with MIC values ranging from 4 µg/mL to 256 µg/mL and above (MIC_50_ = 64 µg/mL). All strains were resistant to cefotaxime, and 35% of the strains showed resistance or reduced susceptibility to ceftazidime. Thirty-nine percent of the strains were identified as carbapenem-resistant *P. aeruginosa* (CRPA) strains, with 37% of the strains showing resistance or reduced susceptibility to both imipenem and meropenem. The MIC values of imipenem ranged from 4 µg/mL to 256 µg/mL and above (MIC_50_ = 16 µg/mL) among the CRPA strains. The AmpC-type β-lactamase gene (*bla*_AmpC_) was detected in 78% of the cephalosporin-resistant strains and 81% of the CRPA. When screened for the presence of metallo-β-lactamase (MBL) genes (*bla*_IMP_ and *bla*_VIM_), only *bla*_VIM_ was detected in 43% of the CRPA strains, while *bla*_IMP_ was absent from all strains.

### *Enterobacteriaceae*—*Enterobacter* spp., *Escherichia coli* and *Klebsiella pneumoniae*

A total of 162 *Enterobacteriaceae* strains, with 54 strains each from *Enterobacter* spp., *E. coli*, and *K. pneumoniae*, were collected and characterized in this study. The *Enterobacter* spp. strains collection is mainly comprised of the species *cloacae* (n = 41) and strains from the *E. cloacae* complex (n = 10), followed by *aerogenes* (n = 1), *asburiae* (n = 1), and *hormaechaei* (n = 1). Majority of the *Enterobacter* spp. (61%) and *K. pneumoniae* (72%) were associated with HA infections. On the contrary, *E. coli* strains were slightly more common from community origins (54%). All *Enterobacteriaceae* strains were screened for their activities against cephalosporins (ceftazidime, ceftriaxone, cefotaxime, cefepime, and cefoxitin), β-lactam combination agents (amoxicillin-clavulanate and piperacillin-tazobactam), aminoglycoside (amikacin), fluoroquinolone (ciprofloxacin), carbapenems (imipenem and meropenem), folate pathway antagonist (sulfamethoxazole-trimethoprim), and lipopeptide (colistin). High rates of MDR were observed in both *E. coli* and *K. pneumoniae*, but the *Enterobacter* spp. remained largely susceptible to the antimicrobial agents tested in this study. Amikacin was the only active antimicrobial agent against all *Enterobacteriaceae* strains.

**Fig. 1:**
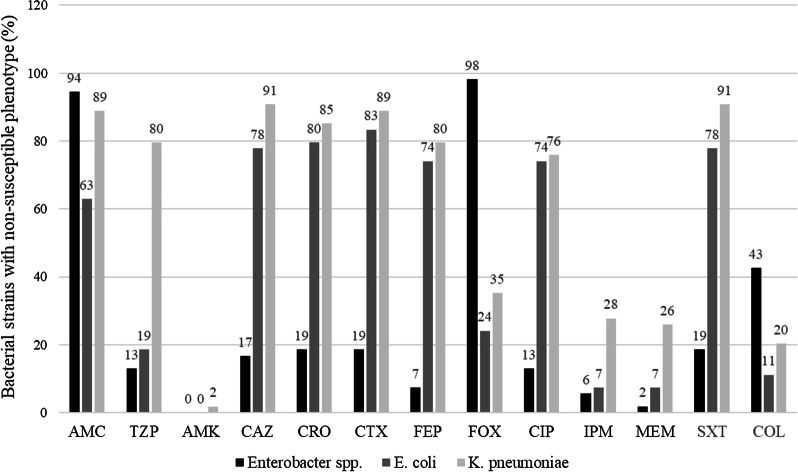
Percentage of non-susceptible *Enterobacteriaceae* strains tested against selected antimicrobial agents. The number on top of each column indicates the percentage value. AMC: amoxicillin-clavulanate; TZP: piperacillin-tazobactam; AMK: amikacin; CAZ: ceftazidime; CRO: ceftriaxone; CTX: cefotaxime; FEP: cefepime; FOX: cefoxitin; CIP: ciprofloxacin; IPM: imipenem; MEM: meropenem; SXT: sulfamethoxazole-trimethoprim; COL: colistin

The *Enterobacter* spp. remained largely susceptible to all antimicrobial agents tested, except for colistin (43% resistance). Colistin resistance was relatively less common in *E. coli* (11%) and *K. pneumoniae* (20%). High rates of resistance to amoxicillin-clavulanate and cefoxitin among the *Enterobacter* spp. were expected due to intrinsic resistance. *E. coli* and *K. pneumoniae* both exhibited high rates of non-susceptibility to cephalosporins, amoxicillin-clavulanate, ciprofloxacin, and sulfamethoxazole-trimethoprim. *K. pneumoniae* strains showed greater resistance to piperacillin-tazobactam and carbapenems compared to the other two organisms. Figure 1 shows a comparison of the non-susceptibility rates of *Enterobacteriaceae* strains against selected antimicrobial agents.

All cefepime-resistant *Enterobacteriaceae* strains showed high-level resistance (MIC_50_ > 256 µg/mL). Non-susceptible *E. coli* exhibited high-level fluoroquinolone and carbapenem resistance (MIC_50_ ≥ 256 µg/mL). Meanwhile, carbapenem-resistant *K. pneumoniae* (CRKP) was highly resistant to imipenem (MIC_50_ > 256 µg/mL). All colistin-resistant strains showed a moderately high level of resistance (8 µg/mL ≤ MIC_50_ ≤ 32 µg/mL). The MIC data of the non-susceptible *Enterobacteriaceae* strains are summarized in Table 4.

**Table 4:** Summary of MIC range and median (MIC_50_) of selected antimicrobial agents for *Enterobacteriaceae* strains with non-susceptible phenotypes

Organism	Cefepime		Ciprofloxacin		Imipenem		Colistin	
	MIC range (µg/mL)	MIC_50_ (µg/mL)	MIC range (µg/mL)	MIC_50_ (µg/mL)	MIC range (µg/mL)	MIC_50_ (µg/mL)	MIC range (µg/mL)	MIC_50_ (µg/mL)
*Enterobacter* spp.	≥ 256	> 256	2—256	8	4—128	4	8—64	32
*E. coli*	8—256	> 256	0.5—256	> 256	2—256	256	4—64	8
*K. pneumoniae*	64—256	> 256	0.5—256	32	4—256	> 256	4—64	32

The most encountered ESBL genes in *K. pneumoniae* were *bla*_SHV_ (94%), *bla*_CTXM-1_ (80%), and *bla*_TEM_ (57%). The majority (80%) of the ESBL-producing *K. pneumoniae* harbored at least two ESBL genes. The MBL gene *bla*_NDM_ was detected in 20% of the *K. pneumoniae*, with one strain harboring two MBL genes (*bla*_NDM_ and *bla*_VIM_). The carbapenemase gene *bla*_OXA-48_ was detected only in *K. pneumoniae* (n = 4). *E. coli* harbored mainly *bla*_TEM_ (56%) and *bla*_CTXM-1_ (37%) and was seen with a less diverse β-lactamase genes profile compared to *K. pneumoniae*. The MBL gene *bla*_NDM_ was detected in a carbapenem-resistant *E. coli* strain. Table 5 summarizes the β-lactamase gene profiles of the *K. pneumoniae* and *E. coli* strains examined in this study.

**Table 5:** Summary of ESBL and carbapenemase gene profiles in *K. pneumoniae* and *E. coli*

β-lactamase gene profile	No. (%) of strains	
	*K. pneumoniae*	*E. coli*
*bla*_SHV_*, bla*_TEM_*, bla*_CTXM-1_*, bla*_NDM_*, bla*_OXA-48_	1 (2)	0 (0)
*bla*_SHV_*, bla*_TEM_*, bla*_CTXM-1_*, bla*_NDM_	9 (17)	0 (0)
*bla*_SHV_*, bla*_TEM_*, bla*_CTXM-1_*, bla*_OXA-48_	2 (4)	0 (0)
*bla*_TEM_*, bla*_CTXM-1_*, bla*_NDM_*, bla*_VIM_	1 (2)	0 (0)
*bla*_TEM_*, bla*_CTXM-1_*, bla*_OXA-48_	1 (2)	0 (0)
*bla*_SHV_*, bla*_TEM_*, bla*_CTXM-1_	16 (30)	0 (0)
*bla*_SHV_*, bla*_CTXM-1_	12 (22)	1 (2)
*bla*_SHV_*, bla*_TEM_	0 (0)	2 (4)
*bla*_TEM_*, bla*_CTXM-1_	1 (2)	12 (22)
*bla*_CTXM-1_	0 (0)	7 (13)
*bla*_NDM_	0 (0)	1 (2)
*bla*_SHV_	11 (20)	0 (0)
*bla*_TEM_	0 (0)	16 (30)

All *Enterobacteriaceae* strains with non-susceptible phenotype to ciprofloxacin were screened for the presence of plasmid-borne fluoroquinolone resistance-conferring genes (*qnr*A, *qnr*B, and *qnr*S). Both *qnr*B and *qnr*S were detected in ciprofloxacin-resistant *E. coli* (10% and 15%), *K. pneumoniae* (61% and 34%), and *Enterobacter* spp. (14% and 43%). A subset of the ciprofloxacin-resistant *K. pneumoniae* (15%) harbored both *qnr*B and *qnr*S. However, there was no indication that the presence of more than one *qnr* gene in the *K. pneumoniae* strain resulted in an increased MIC of ciprofloxacin. Sequence analysis of the quinolone resistance determining region (QRDR) in the DNA topoisomerase II (*gyr*AB) and IV (*par*CE) genes was conducted to identify mutations in the ciprofloxacin-resistant *E. coli* strains. Amino acid substitutions that occurred in the QRDR of each gene are summarized in Table 6. Half of the ciprofloxacin-resistant strains possessed more than one amino acid substitutions in *gyr*A QRDR. The detailed QRDR and *qnr* genes profiles of the *E. coli* strains are shown in Additional file 1: Table S10.

**Table 6:** Amino acid substitution profiles for ciprofloxacin-resistant *E. coli* (n = 40)

Target gene	Amino acid substitution profile*	No. (%) of strains
*gyr*A	Ser-83-Leu; Asp-87-Asn; Leu-102-Ala	4 (10)
	Ser-83-Leu; Asp-87-Tyr; Gly-105-Val	2 (5)
	Ser-83-Leu; Asp-87-Asn	14 (35)
	Ser-83-Leu	6 (15)
*gyr*B	None	37 (93)
*par*C	Ser-80-Ile; Glu-84-Ile	7 (18)
	Ser-80-Ile	16 (40)
*par*E	Ser-458-Ala	2 (5)

*Ala: alanine; Asp: aspartate; Asn: asparagine; Glu: glutamate; Gly: glycine; Ile: Isoleucine; Leu: leucine; Ser: serine; Tyr: tyrosine; Val: valine

### Correlation between AMR trends of the ESKAPEE strains and hospital-acquired infection

Risk factors associated with HA infections caused by each of the ESKAPEE organisms were identified. Logistic regression using forward stepwise selection was undertaken to control the effect of confounding variables. All variables with a significant association with HA infections (p < 0.1) were entered into the logistic regression model. The outcome of the analysis is expressed as odds ratios, Exp(B), indicating the chances of HA infection occurring in the patient. A summary of the correlation analysis results is shown in Table 7.

**Table 7:** Factors associated with hospital-acquired infections

Organism	Risk factor*	P-value	OR	95% CI	
				Lower	Upper
*Enterococcus* spp.	Gender	0.0757	–	–	–
	Ampicillin resistance	0.0757	3.13	0.86	11.37
	Streptomycin resistance	0.01431	–	–	–
*S. aureus*	MDR	0.014	–	–	–
	Clindamycin/Erythromycin resistance	0.014	–	–	–
	Cefoxitin/Oxacillin resistance	< 0.001	7.62	1.36	42.71
	Linezolid/Rifampicin/Vancomycin resistance	< 0.001	–	–	–
	AMR genes	0.02	–	–	–
*K. pneumoniae*	Gender	0.06136	–	–	–
	Race	0.1082	–	–	–
	Ciprofloxacin resistance	0.1	–	–	–
	Imipenem resistance	0.1285	–	–	–
	Colistin resistance	< 0.001	–	–	–
*A. baumannii*	Imipenem/Piperacillin-tazobactam/Colistin resistance	< 0.001	–	–	–
	AMR genes	0.1185	–	–	–
*P. aeruginosa*	Ceftazidime resistance	0.01721	5.04	1.24	20.43
	Cefotaxime resistance	0.05676	–	–	–
	Imipenem resistance	0.1083	–	–	–
	Meropenem resistance	0.0468	–	–	–
	AMR Genes	0.113	–	–	–
*Enterobacter* spp.	Amikacin resistance	0.1025	–	–	–
	Ceftazidime resistance	0.0611	–	–	–
	Ceftriaxone/Cefotaxime resistance	0.0379	7.50	0.87	64.36
*E. coli*	Gender	0.1056	–	–	–
	MDR	0.09166	–	–	–
	Piperacillin-tazobactam resistance	0.09584	–	–	–

*AMR: antimicrobial resistance; MDR: multidrug resistance; OR: odds ratios; CI: confidence interval

## Discussion

We report herein the genotypes and phenotypes of the AMR-ESKAPEE pathogens isolated from a tertiary hospital located in the capital Kuala Lumpur, Malaysia. A total of 378 bacterial strains, with each species represented by 54 strains, were collected over a nine-month study period via the convenient sampling method. All ESKAPEE pathogens were mainly associated with HA infections, except for *E. coli*, whereby slightly more than half of the strains were obtained from community origins. MDR phenomenon was commonly observed among the strains, and the presence of multiple AMR-conferring genes was detected. Most of these AMR strains were isolated from respiratory sites, blood, body fluids, and urine samples.

We observed an increased prevalence of VRE when compared to recent studies within the same geographical region. Moussa and colleagues reported a 4% occurrence of VRE among the 75 enterococci strains obtained in Hospital Kuala Lumpur (HKL), all of which were *E. faecalis* [17]. An earlier study by Daniel et al*.* examining 22 *E. faecalis* strains from Hospital Serdang did not identify any VRE strains [18]. All three hospitals, including UMMC in this study, are located in Klang Valley within close proximity of each other. Previous studies reported a greater prevalence of the *van*A gene among Malaysian VRE strains isolated from various sources, including humans, farmed animals, and even within the natural environment [18, 19]. However, we observed that the *van*B gene occurred more frequently in the VRE strains identified in this study. Both *van*A and *van*B genes are responsible for acquired vancomycin resistance in enterococci, although *van*A confers a higher level of inducible resistance compared to variable levels of inducible resistance conferred by *van*B genes [20]. Indeed, the VRE strains in this study harboring *van*B only showed variable vancomycin MIC values (16 µg/mL to > 256 µg/mL). The greater prevalence of *van*B-harboring VRE in our study site may indicate a possible shift in the predominant VRE genotype in this region. Nonetheless, a further study involving a larger sample size or more diverse sampling sites should be conducted to determine if the observed change in the predominance is a localized or nationwide event.

The enterococci strains in our sample pool showed a slightly lower prevalence of HLAR (39%) compared to a similar study within the same region. A recent study in HKL documented 45–49% HLAR, concerning streptomycin resistance, among the enterococci strains [17]. In the HKL study, the *aac(6′)-Ie-aph(2″)-Ia* AME gene was most common among the HLAR enterococci, accounting for their high level of resistance to gentamicin. The authors reported that the presence of another AME gene, *aph(3′)-IIIa*, could have attributed to the high-level streptomycin resistance among their strains collection. However, our enterococci strains showed a greater prevalence of *ant(4′)-Ia* (22%), followed by *aph(2″)-Ic* (15%), and a total absence of *aph(3′)-IIIa*. The HKL study did not screen for *ant(4′)-Ia*, and none of the strains carried *aph(2″)-Ic* gene [17]. Given that AME genes are responsible for acquired aminoglycoside resistance through horizontal gene transfer, we infer that there might be different lineages of aminoglycoside resistant enterococci currently circulating in this region, each lineage developing resistance through a separate evolutionary pathway.

The association of MDR-MRSA with HA infections and the isolation of mainly drug-susceptible MSSA strains from community origins, as observed in this study, is a common phenomenon among Malaysian *S. aureus* populations [21, 22]. Nonetheless, we observed an occurrence of 28% CA-MRSA, the majority of which were MDR (70%). The increasing prevalence of CA-MRSA has been documented globally, mainly in the Western regions, and gradually replacing the dominant pandemic HA-MRSA clone ST239/ST241-III [23]. The genetically diverse CA-MRSA is known to be a successful colonizer capable of causing a wide range of diseases from mild skin and soft tissue infections to severe systemic infections [23]. This notion is reflected in our CA-MRSA collection from various clinical specimens including pus, blister fluid, blood, sputum, and bronchoalveolar lavage.

We observed simultaneous resistance to cefoxitin and oxacillin among the MRSA strains identified in this study, all of which carrying the *mec*A gene. This finding corresponds with the prediction of *mec*A-mediated methicillin (oxacillin) resistance based on the cefoxitin-resistant phenotype of the strains [15]. Throughout the years, *mec*A-mediated resistance has been the sole mechanism of resistance among MRSA populations in Malaysia [22, 24]. Only until very recently that the presence of the *mec*C gene is documented among livestock-associated MRSA strains isolated in Northern Malaysia, at an alarming rate of 16%, some co-existing with the *mec*A gene [25]. This shows that the novel *mec*C gene has spread beyond Europe and is currently emerging in Malaysia. We recommend screening for the *mec*C gene in future genotyping of clinical *S. aureus* strains to understand the molecular epidemiology and correlated clinical outcomes of this genotype.

Although all *S. aureus* strains examined in this study remained susceptible to vancomycin, the subset of strains (40%) with a relatively higher MIC value (1 µg/mL) within the susceptible range of vancomycin should not be neglected. Reduced susceptibility of *S. aureus* to vancomycin has been increasingly reported worldwide, constituting one of the major global AMR problems [26]. Heterogeneous vancomycin-intermediate *S. aureus* (hVISA) associated with treatment failure has emerged in Malaysian hospitals since 2009 and was increasingly reported during the past decade [27, 28]. Local health authorities should closely monitor vancomycin use in the healthcare settings to slow down, if not completely prevent, the development of resistance to this last-resort drug for the treatment of MRSA infections.

In the hospital diagnostic laboratory, *S. aureus* strains that were identified as erythromycin-resistant are assumed to conform to inducible macrolide-lincosamide-streptogramin B (iMLS_B_) phenotype, i.e. inducible clindamycin resistance. This assumption is essential in the clinical setting, given that iMLS_B_ *S. aureus* shows in vitro susceptibility to clindamycin, hence may lead to therapeutic failure. We observed clindamycin resistance and potential iMLS_B_ in 80% of the MRSA strains, and none in MSSA strains. Similar studies in Malaysia have reported the greater prevalence of clindamycin resistance and iMLS_B_ in MRSA from both clinical (47%) and community (75%) settings [22, 29]. A study conducted in East Coast Malaysia has identified that the iMLS_B_ *S. aureus* predominantly harbored *msr*A gene which confers erythromycin resistance, with the *erm*C gene detected in only a single iMLS_B_ strain [22]. On the contrary, all erythromycin-resistant MRSA strains in our collection harbored the *erm*C gene. This genotype is common in macrolide-resistant MRSA clones in Europe [30]. Therefore, we infer that the iMLS_B_ MRSA from the East and West Coast Malaysia constituted two different clonal lineages. Nonetheless, a further study involving a larger sample size is essential to verify this notion.

MDR *A. baumannii* has been extensively associated with HA infections, often pneumonia, in hospitalized patients especially in the ICUs in Malaysian hospitals [31]. We identified two CA-MDR-CRAB strains isolated from swab and urine specimens of patients admitted to UMMC. Although the CA-MDR-CRAB strains in this study were not associated with life-threatening infections, the presence of MDR *A. baumannii* in the community should not be neglected, as they are capable of causing severe pneumonia in otherwise healthy individuals [32]. The AMR trends and resistance mechanisms of *A. baumannii* in clinical settings have been extensively studied in Peninsular Malaysia over the past thirty years [31]. Nationwide surveillance by the Ministry of Health (MOH) Malaysia has documented a 50–60% resistance rates to carbapenems [33]. However, the frequency of CRAB in hospitals located in Kuala Lumpur, including UMMC, often exceeds the national rates (70–98%) [34, 35]. Similar to other reported studies in this region, the predominant carbapenemase genes identified among the CRAB strains in our collection are *bla*_OXA-23_ and *bla*_OXA-51_ [31, 34]. Additional factors compounding the challenges with AMR include the ubiquitous presence of the ESBL gene *bla*_TEM_ in conjunction with the carbapenemase genes which might have contributed to the relatively high levels of resistance to the antimicrobial agents tested (MIC_50_ ≥ 128 µg/mL). We detected four CRAB strains harboring the acquired ESBL gene *bla*_VEB_. The *bla*_VEB_-carrying *A. baumannii* has been mainly reported in Europe and the Middle East causing nationwide outbreaks and HA infections [36–38]. In the Southeast Asian region, a study on class I integron revealed that the *bla*_VEB_-carrying *A. baumannii* in Thailand is genetically linked to the European clones [39]. Therefore, the emergence of *bla*_VEB_-carrying CRAB strains in Malaysia could be a result of foreign clonal invasion or the exchange of genetic elements between local and global strains.

Approximately one-fifth of the CRAB strains were resistant to colistin, one of the last-resort drugs for the treatment of MDR *A. baumannii* infections. A high rate of resistance to polymyxin (26%) was also documented in a hospital located on the East Coast of Malaysia [40]. Combination therapy using colistin/rifampicin or colistin/carbapenem is recommended for treating MDR *A. baumannii* infections [41]. However, the high rates of carbapenem resistance among Malaysian MDR *A. baumannii* have left us with limited therapeutic options. Studies on the prevalent aminoglycoside resistance mechanisms among Malaysian *A. baumannii* has not been previously reported. We found that the amikacin-resistant CRAB in this study predominantly harbored the *aad*B gene. Interestingly, the *aph*A6 gene that confers amikacin resistance was not detected among the strains [42]. Therefore, amikacin resistance among the CRAB strains could be attributable to other AME genes such as *ant(2′)-Ia*, *aph(3′)-*VIa, *aac(3′)-Ia*, or the upregulation of the efflux pump gene *ade*B [43]. Nonetheless, this notion requires further verification via a thorough genetic study on the aminoglycoside resistance mechanisms.

Similar to *A. baumannii*, *P. aeruginosa* is often associated with HA pneumonia. *P. aeruginosa* is one of the leading nosocomial pathogens in UMMC since the early 2000s [44]. The nationwide AMR surveillance revealed overall ceftazidime, ciprofloxacin, and carbapenems resistance rates of less than 10% among the *P. aeruginosa* populations in Malaysia [33]. When compared to the national surveillance data, the AMR *P. aeruginosa* strains in our study showed higher rates of resistance to the named antimicrobial agents (20–37%). Interestingly, we observed a marked reduction in the AMR rates of *P. aeruginosa* when compared to a study conducted in UMMC approximately 10 years ago, which recorded extremely high rates of resistance to similar antimicrobial agents (74–92%) [45]. Similar high rates of resistance in *P. aeruginosa* were also documented in another two hospitals located within the same geographical location, at around the same period [46, 47]. An even earlier UMMC study in 2005 showed only 10–11% of *P. aeruginosa* were resistant to the same agents [48]. The fluctuation in the AMR rates of *P. aeruginosa* within the past two decades could have been a result of competition between antimicrobial stewardship and the microevolution of the pathogen. Effective antimicrobial stewardship programs (ASPs) might successfully reduce the rates of resistance, but the organism can develop different resistance mechanisms through horizontal gene transfers or mutations, or a different resistant clone might gain dominance in the same setting.

The persistence of CRPA has been a long-term nosocomial problem in UMMC. Multiple studies have been conducted intermittently by different research groups to understand the molecular epidemiology of CRPA in this institution. CRPA carrying an acquired MBL gene *bla*_IMP-7_ has emerged in 1999, isolated from the peritoneal fluid of an 8-year-old child [49]. A retrospective study that screened CRPA strains isolated between 2005–2008 has detected four MBL gene variants, namely *bla*_IMP-7_ (13%), *bla*_IMP-4_ (2%), *bla*_VIM-2_ (19%), and *bla*_VIM-11_ (1%) [50]. DNA fingerprinting results revealed that the *bla*_IMP_- and *bla*_VIM_-carrying CRPA constituted two distinctive clones [51]. The detection of only *bla*_VIM_-carrying CRPA in the current study suggested the evolutionary success of this clone, indicated by its decade-long persistence in this institution. A recent study conducted in a hospital located in Johor (Southern Malaysia) has discovered MBL gene variants that were not previously reported in Malaysian CRPA, namely *bla*_IMP-1_ and *bla*_NDM-1_ [52]. Besides MBL genes, the ubiquitous AmpC-type β-lactamase gene present in the cephalosporin-resistant and CRPA (in this study) could have contributed to the resistance phenotypes [53].

MDR *Enterobacteriaceae* is one of the greatest public health threats worldwide, with an ever-increasing trend of ESBL-producing strains at 5.4% per annum [54]. Multiple studies have investigated the AMR mechanisms of ESBL-producing *E. coli* and *K. pneumoniae* in Malaysia. The most encountered ESBL genes among these two organisms are *bla*_TEM_, *bla*_SHV_, and *bla*_CTXM_ [55, 56]. Similar observations were made in our study. Besides, the *K. pneumoniae* strains examined in this study harbored a more diverse range of ESBL genes compared to *E. coli*. Clinical strains of the *Enterobacteriaceae* family in Malaysia has long been known to exchange AMR determinants via horizontal gene transfer, for instance, the transfer of R-plasmids [57]. Co-occurrence of *K. pneumoniae* strains harboring multiple ESBL genes with other *Enterobacteriaceae* organisms in the hospital environment greatly enhances the development of cephalosporin resistance among the nosocomial pathogens [58].

Carbapenem-resistant *Enterobacteriaceae* (CRE) is a growing public health threat in most parts of the world [59]. We observed a higher frequency of carbapenem resistance among *K. pneumoniae* compared to *E. coli* and *Enterobacter* spp. Both carbapenem-resistant *K. pneumoniae* (CRKP) and *E. coli* (CREC) showed high-level resistance to imipenem (MIC_50_ ≥ 256 µg/mL). The majority of the CRE (82%) were simultaneously resistant to both imipenem and meropenem. These genes were more common among the CRKP (*bla*_NDM_ and *bla*_VIM_), with only one CREC carried a *bla*_NDM_ gene. The higher rate of carbapenem resistance in *K. pneumoniae* with a predominantly *bla*_NDM_ genotype is a common phenomenon in Malaysian hospitals [60, 61]. The *bla*_NDM_-carrying CRKP currently circulating in Malaysia could have been imported from India [62]. We detected the presence of *bla*_VIM_ in CRKP, which had not been previously identified among *K. pneumoniae* in Malaysia. Besides MBL genes, the OXA carbapenemase gene that is most prevalent among CRE, *bla*_OXA-48_, was also found in our CRKP [59]. Strains harboring these two carbapenemases (NDM and OXA) appeared common among the CRE populations in Southeast Asia [63]. Based on our analysis, CRKP carrying only *bla*_OXA-48_ had a lower MIC of imipenem (MIC_IPM_ = 4 µg/mL) compared to strains carrying the MBL gene(s) (MIC_IPM_ > 256 µg/mL). The ability of the MBL genes to confer high-level carbapenem resistance could explain the shift from a predominantly *bla*_OXA-48_- to *bla*_NDM_-carrying CRKP population in our institution from 2013 to the current [64].

Increasing resistance to colistin, the last-resort drug for treating CRE infections, is currently on the rise on a global scale but under-reported in Southeast Asian countries [63]. To date, the MOH Malaysia has only been monitoring colistin resistance among *P. aeruginosa* in local hospitals, recording a slightly increased rate of resistance in 2018 within five years from 2013 (from 0.43% to 0.6%) [33]. We recorded the highest rate of colistin resistance in *Enterobacter* spp. (43%), followed by *K. pneumoniae* (20%) and *E. coli* (11%). This observation is in agreement with the global surveillance that identified *Enterobacter* as the most common *Enterobacteriaceae* species that developed colistin resistance in clinical settings [65]. Among the colistin-resistant strains in this study, four were CRKP (4 µg/mL ≤ MIC_COL_ ≤ 32 µg/mL) and two were CR-*Enterobacter* (MIC_COL_ > 64 µg/mL). Reports on colistin-resistant *Enterobacteriaceae* are rarely documented in Malaysia. There were only a few studies that reported colistin resistance among *K. pneumoniae*, with a higher incidence in zoonotic strains from swine farms compared to clinical strains [55, 66, 67]. We observed emerging colistin resistance among the CRE in the clinical setting. Therefore, increasing efforts to strengthen surveillance of this phenotype in local hospitals is essential to better understand the extent and impact of this public health problem.

Another major AMR problem associated with *Enterobacteriaceae* is fluoroquinolone resistance. Two important genetic mechanisms that confer fluoroquinolone resistance to *Enterobacteriaceae* are chromosomal mutation in the QRDR of the DNA topoisomerases and plasmid-borne *qnr* genes [68]. We detected *qnr*B and *qnr*S in the ciprofloxacin non-susceptible strains, with the highest frequency in *K. pneumoniae*, some of which harbored both *qnr* genes. Constant absence of *qnr*A has been observed among *Enterobacteriaceae* in Malaysia [69, 70]. The regular use of quinolone antibiotics in our country might not favor the selection of strains with *qnr*A as it only confers low-level resistance to quinolone [71]. Mutations in the QRDR of *gyr*A and *par*C are common among *Enterobacteriaceae* isolated worldwide [72]. We identified amino acid substitution hotspots at Ser-83 and Asp-87 in *gyr*A, and Ser-80 and Glu-84 in *par*C, generally conferring a higher level of ciprofloxacin resistance compared to wild type strains. Mutations at these sites and/or the presence of *qnr*B or *qnr*S seemed to be the common phenotypes of fluoroquinolone-resistant *Enterobacteriaceae* in this region [69, 70, 73]. We detected two novel mutations in the *gyr*A QRDR of *E. coli*, namely Leu-102-Ala and Gly-105-Val, not reported in previous literature [74]. Similar to previous reports, mutations were rare in *gyr*B and *par*E among our *E. coli* strains [69, 70].

We identified four factors that were linked with an increased risk of HA infection: ampicillin-resistant *Enterococcus* spp. (3.13 times higher chance), MRSA (7.62 times), ceftazidime-resistant *P. aeruginosa* (5.04 times), and ceftriaxone/cefotaxime-resistant *Enterobacter* spp. (7.5 times). In general, resistance to β-lactam antibiotics among the ESKAPEE organisms, except for *S. aureus*, is a significant predictor for patients’ chances of acquiring infections in hospital settings. A review of the current antimicrobial stewardship program in the hospital should be conducted to assess the use of these antimicrobial agents in the institution. Enhancement of current infection control practices should be made concurrently to reduce the rate of HA infections caused by these AMR organisms.

## Conclusions

In summary, data obtained in this study may provide valuable insight into the AMR trends and the major genetic mechanisms that conferred resistance to the ESKAPEE pathogens isolated from a tertiary hospital located in the capital city Kuala Lumpur, in the central region of Peninsular Malaysia. Our study included only a limited number of bacterial strains representing each of the ESKAPEE species, hence may not completely reflect the actual AMR prevalence of these organisms in this region. Nonetheless, this is the first report in Malaysia that collectively assessed the phenotypic and genotypic characteristics of the seven bacterial species that are of great public health importance in healthcare settings. Our findings may serve as the cornerstone for future works on the molecular epidemiology of ESKAPEE pathogens in this region. Furthermore, given that AMR is no longer a rare phenomenon in local settings, our selective evaluation of AMR-ESKAPEE strains provided greater insight into the MDR phenomenon and the genetic drives that gave rise to the common AMR phenotypes of these organisms. This information may contribute to making better-informed decisions on appropriate antibiotic prescription plans and infection control strategies in local hospitals.

## Supplementary Information

**Additional file 1.** PCR primers and conditions used for the identification of ESKAPEE strains (Table S1) and the characterization of AMR genes profile of each bacterial species (Table S2), detailed AMR features of the ESKAPEE strains (Table S3-S9), and QRDR mutations and qnr genotypes of ciprofloxacin non-susceptible *E. coli* strains (Table S10).

## Data Availability

All data generated or analyzed during this study are included in this published article [and its supplementary information files].

## Abbreviations

AME: Aminoglycoside-modifying enzyme
AMR: Antimicrobial resistance
ASP: Antimicrobial stewardship program
CA: Community-acquired
CI: Confidence intervals
CLSI: Clinical and Laboratory Standards Institute
CRAB: Carbapenem-resistant *A. baumannii*
CRE: Carbapenem-resistant *Enterobacteriaceae*
CRKP: Carbapenem-resistant *K. pneumoniae*
CRPA: Carbapenem-resistant *P. aeruginosa*
DNA: Deoxyribonucleic acid
ESBL: Extended-spectrum β-lactamase
ESKAPEE: *Enterococcus* Spp., *Staphylococcus aureus, Klebsiella pneumoniae, Acinetobacter baumannii, Pseudomonas aeruginosa, Enterobacter* spp., and *Escherichia coli*
HA: Hospital-acquired
HAI: Hospital-acquired infections
HCAI: Healthcare-associated infection
HGT: Horizontal gene transfer
HKL: Hospital Kuala Lumpur
hVISA: Heterogeneous vancomycin-intermediate *S. aureus*
ICU: Intensive care unit
iMLS_B_: Inducible macrolide-lincosamide-streptogramin B
MBL: Metallo-β-lactamase
MDRO: Multidrug-resistant organism
MIC: Minimum inhibitory concentration
MRSA: Methicillin-resistant *S. aureus*
MSSA: Methicillin-susceptible *S. aureus*
OR: Odds ratios
PCR: Polymerase chain reaction
QRDR: Quinolone resistance determining region
UMMC: University of Malaya Medical Centre
VRE: Vancomycin-resistant enterococci
WHO: World Health Organization
